# Advances in the Management of Spontaneous Coronary Artery Dissection (SCAD): A Comprehensive Review

**DOI:** 10.31083/j.rcm2509345

**Published:** 2024-09-24

**Authors:** Arianna Morena, Federico Giacobbe, Ovidio De Filippo, Filippo Angelini, Francesco Bruno, Stefano Siliano, Giuseppe Giannino, Veronica Dusi, Matteo Bianco, Carloalberto Biolé, Ferdinando Varbella, Enrico Cerrato, Fabrizio D’Ascenzo, Gaetano Maria De Ferrari

**Affiliations:** ^1^Division of Cardiology, Cardiovascular and Thoracic Department, Città della Salute e della Scienza, 10126 Torino, Italy; ^2^Division of Cardiology, Department of Medical Sciences, University of Turin, 10124 Torino, Italy; ^3^Division of Cardiology, A.O.U San Luigi Gonzaga, 10043 Orbassano, Italy; ^4^Interventional Cardiology Unit, Rivoli Infermi Hospital, 10098 Torino, Italy; ^5^Interventional Cardiology Unit, San Luigi Gonzaga University Hospital, 10043 Orbassano, Italy

**Keywords:** spontaneous coronary artery dissection (SCAD), acute coronary syndrome (ACS), women, pregnancy-associated, fibromuscular dysplasia, myocardial infarction, antiplatelet therapy, percutaneous coronary intervention (PCI)

## Abstract

Spontaneous coronary artery dissection (SCAD) is a rare but significant cause of acute coronary syndrome (ACS), primarily affecting young women, often during pregnancy. Despite its rarity, SCAD poses challenges due to limited evidence on management strategies. This review examines the current state of art of SCAD management, integrating interventional and clinical insights from recent studies. The epidemiology of SCAD is related to its elusive nature, representing only a small fraction of ACS cases, while certainly underestimated. Proposed risk factors include genetic, hormonal, and environmental influences. Angiographic classification may help in SCAD diagnosis, but confirmation often relies on intracoronary imaging. Conservative management constitutes the primary approach, showing efficacy in most cases, although optimal antiplatelet therapy (APT) remains debated due to bleeding risks associated with intramural hematoma. Revascularization is reserved for high-risk cases, guided by angiographic and clinical criteria, with a focus on restoring flow rather than resolving dissection. Interventional strategies emphasize a minimalist approach to reduce complications, utilizing techniques such as balloon dilation and stent placement tailored to individual cases. Long-term outcomes highlight the risk of recurrence, necessitating vigilant follow-up and arrhythmic risk assessment, particularly in patients presenting with ventricular arrhythmias. In conclusion, SCAD management always represents a challenge for the physician, both from a clinical and interventional point of view. Recent clinical evidence and a multidisciplinary approach are vital for optimizing patient outcomes and preventing recurrence. This review offers a concise framework for navigating the complexities of SCAD management in clinical practice and proposes an algorithm for its management.

## 1. Introduction

Spontaneous coronary artery dissection (SCAD) represents a rare cause of acute coronary syndrome (ACS) of nonatherosclerotic origin, characterized by compression of the coronary lumen by a blood-containing false lumen, which may be generated by an intimal flap (“inside-out”) or intramural hematoma due to vasa vasorum hemorrhage (“outside-in”). The low incidence and tendency to underdiagnose the condition results in little evidence about the medical and interventional treatment of SCAD. Failure in its detection can result in inappropriate interventions with dangerous complications leading to significant morbidity and mortality [1, 2]. In this review, the state of the art on SCAD management will be analyzed in detail both on the interventional and clinical side, according to the latest evidences provided by clinical studies.

### 1.1 Epidemiology and Risk Factors

The incidence and the true prevalence of SCAD remain uncertain because the condition remains frequently undiagnosed; currently SCAD is estimated to represent 2.1% of all patients presenting with ACS [3]. SCAD mostly affects young women (average age of onset 44–55 years) [3], especially during pregnancy (up to 43% of peripartum myocardial infarctions) [4].

These patients usually present few or no conventional risk factors for atherosclerosis, nevertheless disease-specific causes are not well-known. Some studies have hypothesized a combination of genetic, hormonal, and environmental factors such as emotional distress or extreme physical activity, pregnancy and postpartum, systemic diseases as fibromuscular dysplasia (FMD) or connective tissue disorders (Ehlers-Danlos syndrome, Marfan syndrome, etc.), and cigarette smoking [5, 6]. The predominant female population, precipitating factors, clinical presentation, restitutio ad integrum and significant recurrence risk are also shared with other singular conditions such as Tako-Tsubo Syndrome (TTS) [7, 8, 9]. In particular, as TTS typically occurs after a relevant emotional trigger such as SCAD, cases of concomitant SCAD and TTS presentation are reported in the literature [10] and may further lead to SCAD underdiagnosis as macroscopic features of TTS are often more easily recognizable.

### 1.2 Angiographic Classification

**Fig. 1.**
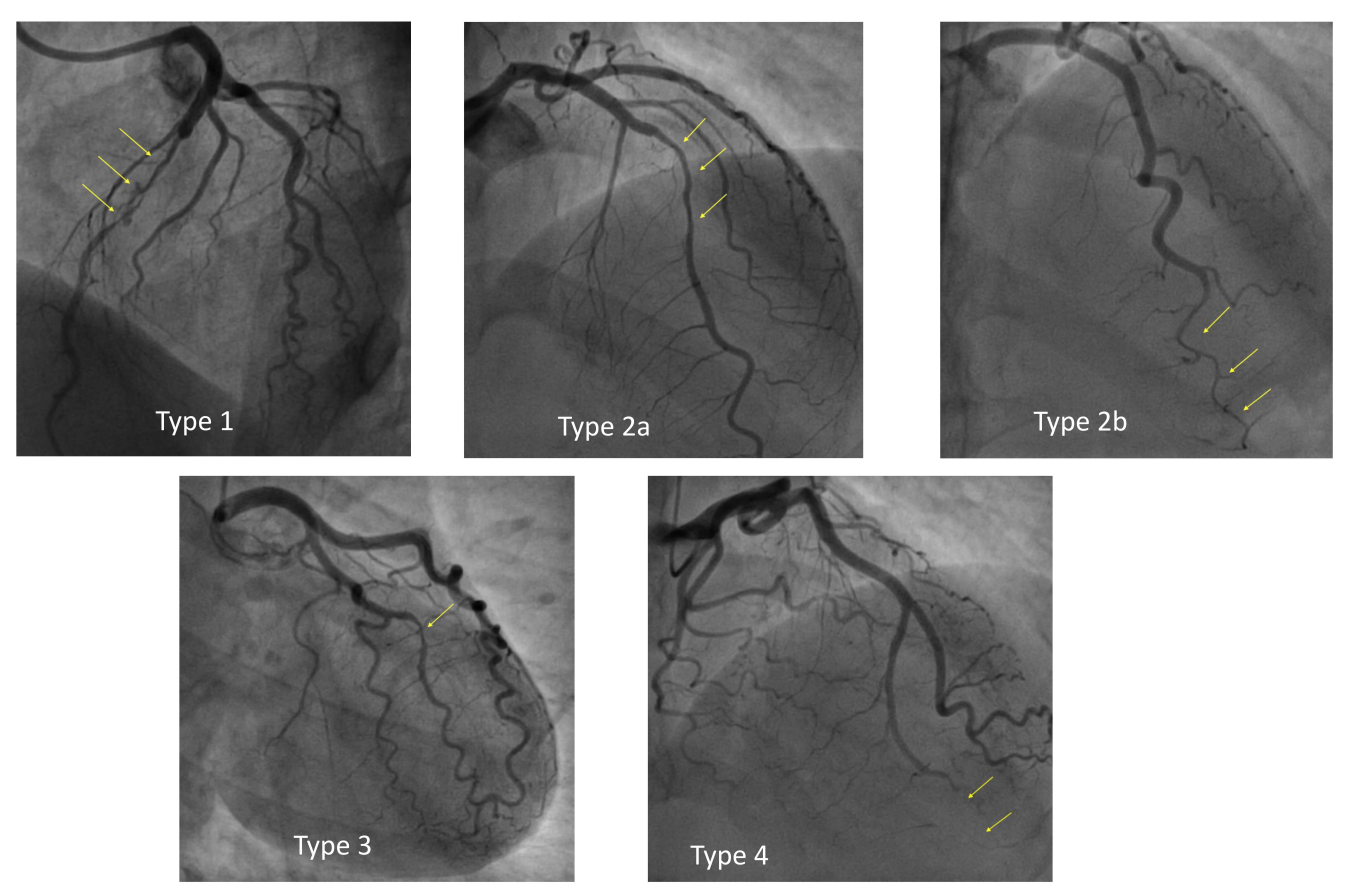
**Angiographic classification of spontaneous coronary artery dissection (SCAD)**.

According to the classification proposed by the Canadian group and adopted by the consensus panel of the European Society of Cardiology, SCAD can be classified angiographically into the following categories [11, 12] (Fig. 1):

- Type 1: double-track image due to contrast stagnation in the false lumen—this represent the easiest pattern to interpret and is pathognomonic of SCAD, but it occurs in only 29% of SCADs.

- Type 2: presence of long narrowing of the vessel lumen, usually >20 mm; represents the most frequent pattern of SCAD, about 67%. Type 2 is further divided into:

- 2a: presence of distal restoration of the native coronary vessel caliber;

- 2b: extension of intramural hematoma up to the distality of the vessel with terminal “rat-tail” appearance.

- Type 3: focal narrowing of the lumen, indistinguishable from atherosclerotic lesions—about 4% of SCADs.

- Type 4: total vessel occlusion; its diagnosis is difficult as it can mimic complete thrombotic occlusion.

In some cases, there may be co-existence of multiple patterns in different coronary arteries as well as at different levels of the same coronary artery. The diagnosis is not immediate and is suspected on the basis of other clinical features (such as the absence of obvious atherosclerosis and the patient’s risk profile), angiographic characteristics and on the use of intracoronary imaging such as intravascular ultrasound (IVUS) and optical coherence tomography (OCT). The intravascular imaging technique used to confirm the diagnosis and to guide a possible revascularization procedure can be either IVUS or OCT indifferently, depending on the operator’s experience. However, some advantages/disadvantages of each method should be considered.

Different study groups have reported an increased risk of reinfarction and unscheduled percutaneous coronary intervention (PCI) in SCAD types 2a and 3 [13, 14]. This association appears to be related to the presence of an intramural hematoma that has not evolved with the creation of a dissecting flap: therefore, these two subtypes are potentially more unstable and correlated with higher event rates during follow-up.

Although SCAD diagnosis and classification are based on coronary angiography, the use of other imaging tools such as coronary computed tomography angiography (CCTA) is increasingly recommended. Indeed, CCTA may avoid complications related to an invasive approach and is usually preferred for screening coronary disease in patients at low cardiovascular risk with atypical symptoms like young women, who represent the typical population affected by SCAD [15, 16, 17]. Most concerns arise from little evidence on real sensitivity and specificity of the tool in this setting, as it may vary according to the type of dissection and to the segment involved [18, 19]. However, some authors have proposed its use also for follow-up to control the healing of the vessel, especially in cases of conservative management [20, 21].

## 2. Management 

Due to the rarity of the disease, there is still lack of consensus concerning the best treatment for SCAD: few studies have reported outcomes of patients conservatively managed or treated with revascularization [22, 23, 24, 25], nevertheless, no randomized trial is available, and the predictors of success for each of the therapeutic approaches are currently debated.

According to the latest European Society of Cardiology (ESC) guidelines on ACS [26] the preferred treatment is the conservative one whenever possible (i.e., in presence of a clinical stability): this is warranted by several studies demonstrating a high rate of dissection healing in the first months after the acute event and of complications related to revascularization strategy [27, 28]. Notably, ACS guidelines in cases of SCAD with associated symptoms, signs of ongoing myocardial ischemia, a large area of myocardium in jeopardy, and reduced antegrade flow suggest performing PCI in class I, level of evidence C.

In this review we present all available evidences on SCAD treatment and propose a therapeutic algorithm to deal with this challenging disease.

## 3. Medical Therapy 

There is no available evidence given by randomized controlled trials on medical treatment in SCAD, despite ongoing studies that will better clarify this aspect [29]. According to the latest data the best treatment of SCAD consists of a conservative approach, which is effective in up to 80% of patients [22, 23, 30]. A retrospective analysis showed an increased deviation towards conservative management by 2019 (89%) when compared to 2013 (35%), which determines the positive impact of this strategy [31]. SCAD, in fact, tends to heal spontaneously and revascularization is hampered by a high rate of complications and worse long-term prognosis. Furthermore, revascularization has no preventive effect on SCAD recurrences, which tend to occur in branches other than those involved at the first event [30, 31, 32, 33].

In conservatively managed cases, a major issue concerns the antiplatelet strategy to be administered as large debate comes from the use of drugs which may cause or worsen bleeding in a condition which is primarily determined by an intramural hematoma. The latest ESC guidelines recommend the same pharmacological treatment as other ACS patients, regardless of the pathophysiology underlying SCAD [26]. However, many questions remain open and have to be clarified in future. The rationale for dual antiplatelet therapy in SCAD conservatively managed is firstly supported by evidence in OCT studies of high-grade stenosis given by true luminal thrombosis, despite the uncommon frequency [34, 35]. In addition, the exposure of blood constituents to prothrombotic submatrix and abnormal shear stress forces as well as the narrowing of the vessel itself due to the ACS-related inflammatory response could further aggravate vessel thrombosis leading to worsening ischemia [36]. Hence, dual antiplatelet therapy (DAPT) is advocated at least for the acute phase [37], though no clear recommendations are given by guidelines or position papers, which merely recommend clopidogrel in place of ticagrelor or prasugrel and do not differentiate between SCAD and conventional ACS in terms of DAPT length [11, 26].

The only evidence available in the literature on antiplatelet therapy in SCAD conservatively managed was given by the results of the Italian-Spanish registry DIssezioni Spontanee COronariche (DISCO) on SCAD [13, 24]. In this cohort, most patients were discharged with DAPT (66.3%), which was associated with a significant increase in major cardiovascular events (defined as the composite of death from all causes, nonfatal myocardial infarction (MI), unplanned PCI: 18.9% vs 6.0%, hazard ratio 2.62, *p* = 0.013). This result was driven by an excess of nonfatal infarctions and unplanned PCI in the early phase of disease, almost all within the first month. A possible explanation for this phenomenon may be found in the physiopathological substrate of SCAD, as the intramural hematoma could be expanded by an increased platelet inhibition with the consequent worsening of ischemia.

This hypothesis could also support the finding that the angiographic pattern of type 2a and 3 (corresponding to a confined hematoma compressing the true lumen) resulted as an independent predictor of adverse events, differently to type 1, where the dissection appears more evident but the evident communication between true and false lumen probably prevents further extension of the hematoma. Considering the results of this study, the routine use of DAPT in conservatively managed patients with SCAD, although suggested by guidelines, should not be recommended; unfortunately, no recommendations can be made about the use of a single antiplatelet therapy (SAPT) or no antiplatelet at all, as there are no data in the literature comparing these two options. For this reason, a randomized clinical trial (BA-SCAD trial) that tries to assess the clinical efficacy of the 2 most widely used pharmacological therapeutic strategies in patients with SCAD in clinical practice is ongoing, namely, to assess the role of beta-blockers and different antiplatelet regimens (short [1 month] duration of SAPT vs DAPT for 1 year) in these patients [29].

Patients treated with stents should receive guideline-based DAPT followed by life-long therapy with aspirin; whereas in patients conservatively managed, the type and duration of antiplatelet therapy should be chosen on a case-by-case scenario. Long-term use may be preferred in patients with FMD or evidence of atherosclerosis [38, 39]. Shorter durations (3–12 months) may be reasonable in patients with heavy menstrual bleeding or those at a high risk of bleeding complications [40].

Data on the prevention of SCAD recurrence are scant; the only available evidence shows that the use of beta-blockers and adequate blood pressure control can reduce the risk of recurrence [41]. The use of angiotensin-converting enzyme (ACE) inhibitors and angiotensin receptor blockers (ARB) in SCAD is recommended according to the guidelines on acute myocardial infarction with (STEMI) or without (NSTEMI) ST-segment elevation and heart failure [26, 42] and to improve hypertension control.

The other drugs commonly used in acute coronary syndromes, e.g., statins, nitrates, and ranolazine, have no evidence for or against [41, 43] and therefore should not be prescribed routinely, but used upon treating physicians’ judgement based on the clinical scenario or indications other than SCAD. In the cohort presented by Tweet *et al*. [22] statin used was associated with a higher rate of SCAD recurrence, however this finding was hampered by low numerosity and an interaction between statin use and time enrollment and was not confirmed in subsequent studies [5, 11]. To date, no study has demonstrated a positive or harmful effect of statins after SCAD: despite their intuitive pleiotropic effect on inflammation and angiogenesis [44], no significant clinical benefit was associated with statins in addition to doubts concerning the low density lipoprotein (LDL) target being reached and adherence to the treatment [36, 43, 45].

## 4. Interventional Strategy 

**Fig. 2.**
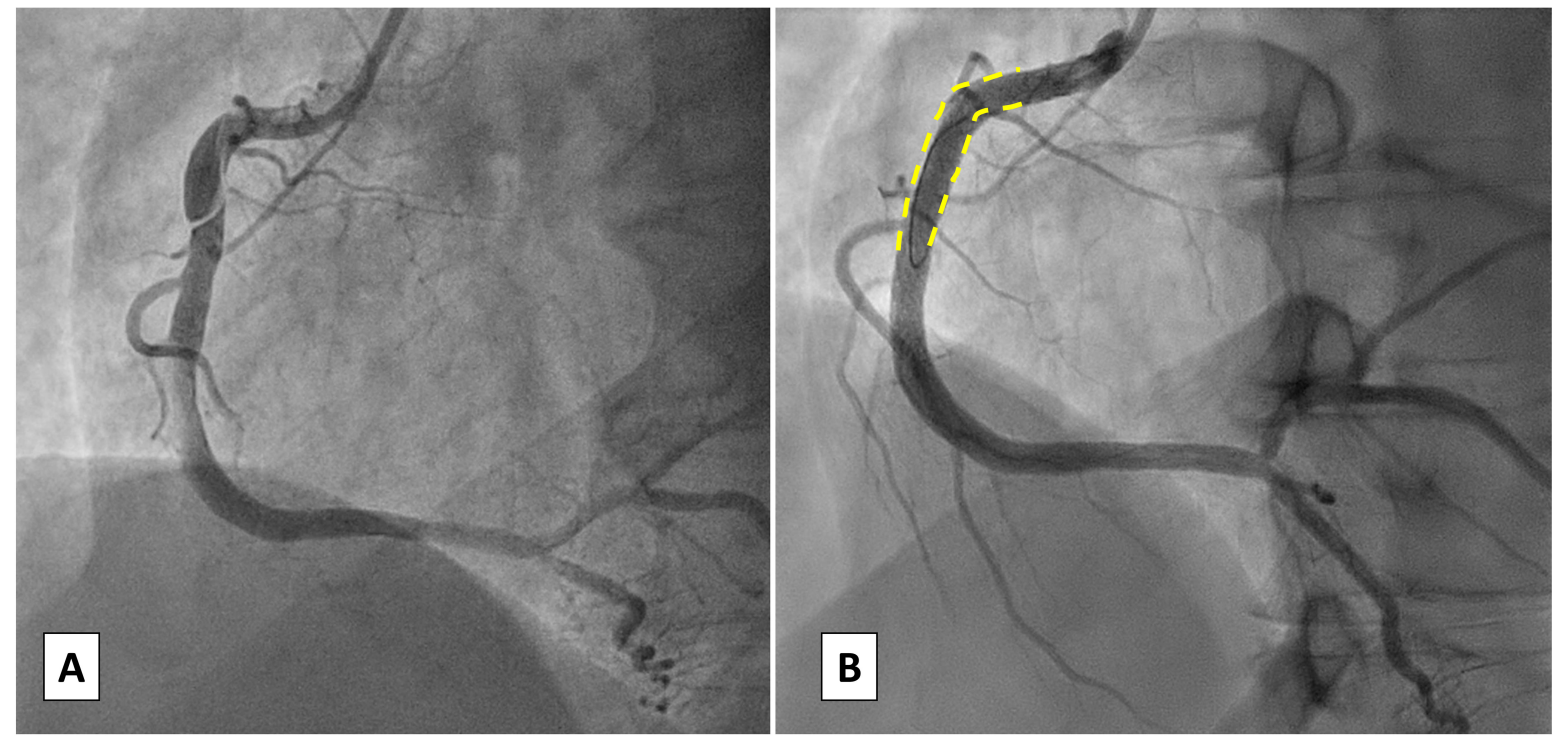
**Case 1: Woman, 56 yo, presenting with acute myocardial infarction without ST-segment elevation (NSTEMI)**. (A) Angiographic presentation (type 1) SCAD of the proximal right coronary artery (RCA) with TIMI 1 flow. (B) Percutaneous coronary intervention (PCI) with implantation of a single Everolimus Eluting Stent due to the angiographic involvement of the proximal segment of RCA. TIMI, thrombolysis in myocardial infarction.

Due to the increased risks of complications and suboptimal results, revascularization should be considered only in high-risk patients, defined according to angiographic and clinical characteristics [46]: persistent chest pain, persistent ST-segment elevation, hemodynamic or electrical instability, proximal location or multiple dissections, left main (LM) dissection, TIMI (thrombolysis in myocardial infarction) 0 and 1 coronary flow. In most cases an interventional treatment is discouraged according to the principle “conservative whenever possible”, yet, in most cases angiographic appearance of multivessel SCAD as well as dissections in proximal segments may lead the operator to perform PCI [47, 48] (Fig. 2).

When the option of percutaneous revascularization is considered, a minimalist approach should be followed: the goal of PCI in SCAD should be to restore flow, not to resolve the dissection, which in most cases will heal on its own [49].

**Fig. 3.**
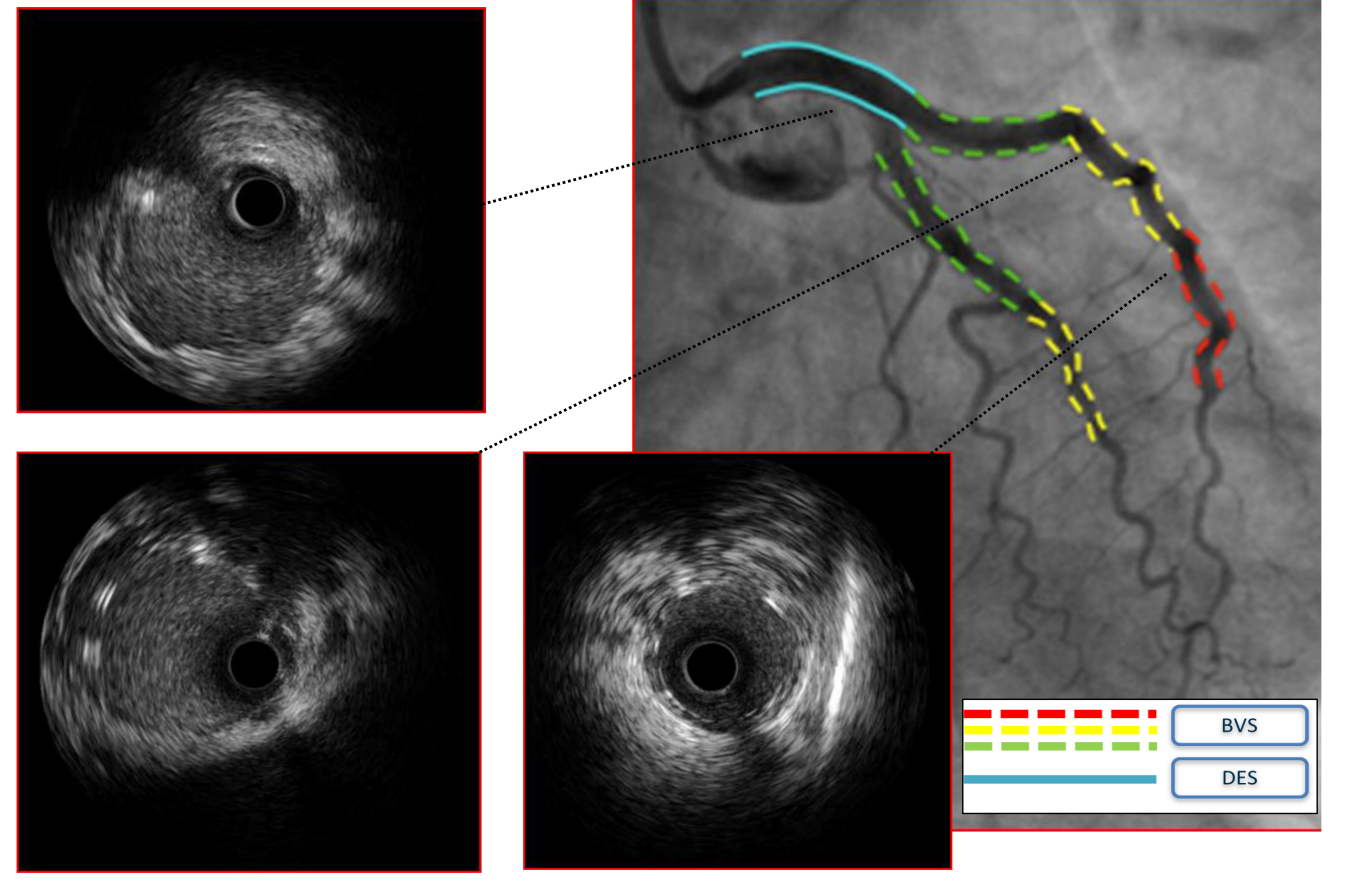
**Case 2: Man, 51 yo, presenting with STEMI with evidence of SCAD of the left main involving also proximal left anterior descending artery (LAD) and circumflex artery (Cx), confirmed at intravascular ultrasound (IVUS) imaging**. Given the young age and the need for an urgent interventional treatment a hybrid approach with bioresorbable vascular scaffolds (BVS) and single drug-eluting stent (DES) on left main (LM) was adopted.

As most patients are young women without concomitant atherosclerotic disease, the use of stent sparing techniques may be preferable in order to avoid modifications of the coronary vessel physiology. As time goes by more and more evidence mainly from case reports or small observational cohorts appears in literature for SCAD in exactly the same way as for traditional angioplasty or peripheral interventions [50, 51]. In this sense the use of bioresorbable scaffolds (BRS) or a hybrid approach with bioresorbable scaffolds and drug eluting stents (BRS-DES) may be considered [52, 53, 54] to avoid long stenting (Fig. 3). In addition, balloon only strategies such as a cutting or drug coated balloon (DCB) may be performed. A cutting balloon may be considered for focal lesions, preferably in a proximal location to drain the intramural hematoma [55, 56]. In a recent review of 32 published cases [57] a cutting balloon resulted a favorable and safe strategy: TIMI 3 flow was restored in almost 85% of cases, despite requiring additional stenting in 37.5%. However, in literature case reports of cutting balloon strategies often report short follow-up length, thus only limited evidence is available on the effectiveness of this approach. Regarding drug coated balloons, scarce data are available and stem from experiences of these tools in iatrogenic dissection healing. The employment of DCB with longer balloon inflation may be considered in case of confirmed intimal tear dissection (“inside-out” mechanism) [58].

PCI may be conducted with only guidewire passage and undersized balloon expansion at low atmospheres to facilitate the creation of fenestrations of the hematoma with its emptying and restoration of flow in the true lumen; for the same purpose, the use of a cutting and scoring balloon has a role in this sense [56].

The use of non-polymeric, low tip load guidewires with good torque control is recommended to facilitate the wiring of the true lumen, avoid post-dilatation and inflations at high atmospheres.

As in most cases of SCAD, patients present with an intrinsic vascular fragility, the risk of iatrogenic dissection is higher than in other cases, thereby a deep catheter intubation should be avoided [28]. Likewise, extreme attention should be paid to the management of vascular access: as SCAD patients’ vessels are more fragile and prone to dissection, the American Heart Association recommends preferring femoral access or using radial but with great caution, based on published studies in which the latter was associated with an increased risk of iatrogenic catheter dissection [5, 59]. If radial access is used, special care should be taken to avoid deep catheter intubation, noncoaxial placement, and high-pressure contrast injection. However, in view of the proven experience in Europe inherent in the use of radial access, we think it is appropriate to suggest the use of arterial access with which the operator feels more confident.

**Fig. 4.**
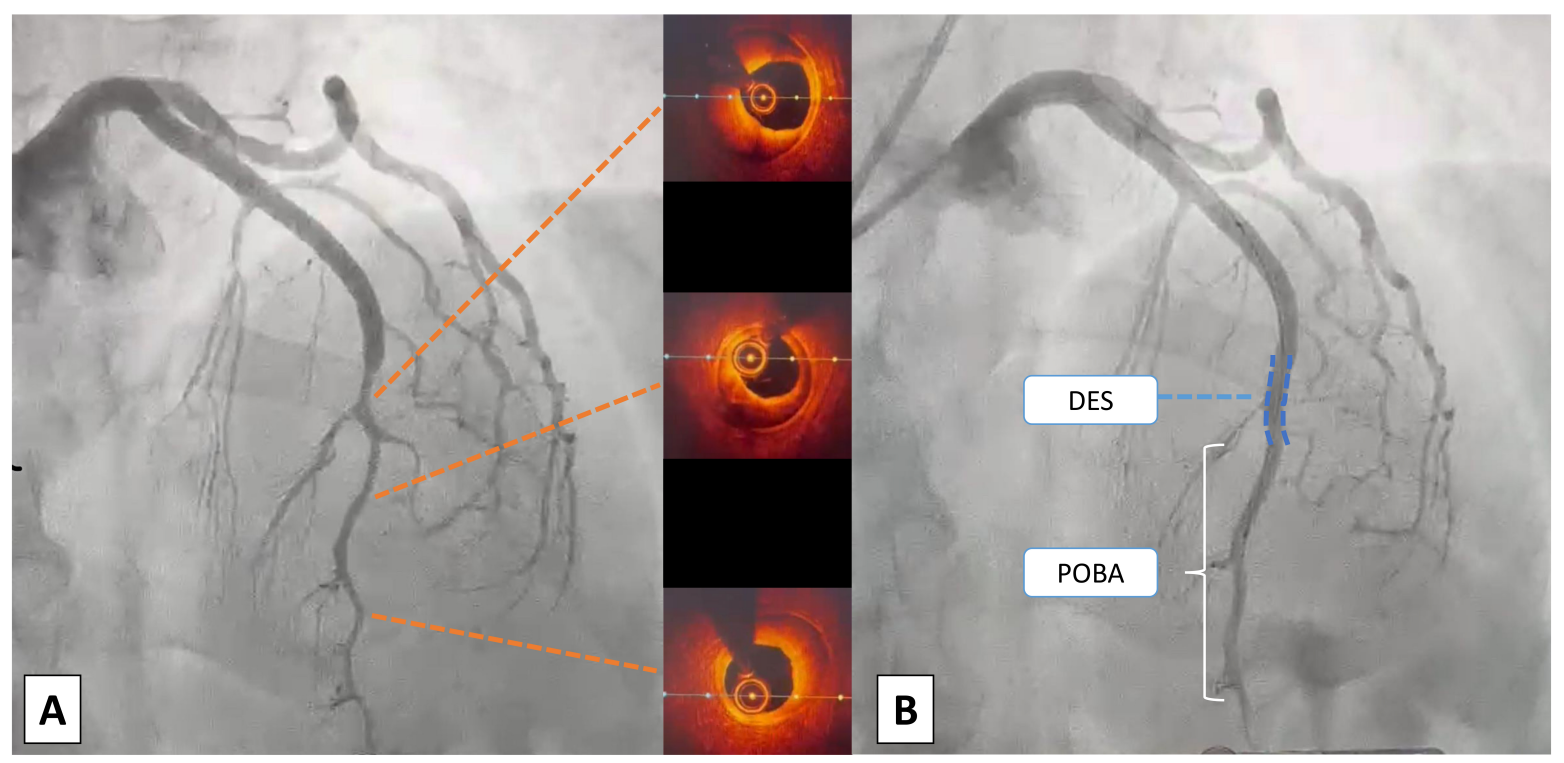
**Case 3: Woman, 52 yo, presenting with NSTEMI, on the way to the emergency department an episode of ventricular fibrillation treated with a single direct current (DC) shock**. On admission persistence of chest pain unresponsive to nitrate therapy, normal ECG and ipokinesia of the antero-lateral wall at echo: urgent coronary angiography was indicated. (A) At coronary angiography SCAD of medium and distal LAD, confirmed with intracoronary imaging using optical coherence tomography (OCT) with evidence of outside-in mechanism (absence of intimal flap). (B) Due to the clinical instability (ventricular fibrillation (VF) before admission and refractory pain) an interventional strategy was chosen: a short single drug-eluting stent (DES) was deployed to fix the proximal cap of the dissection to prevent retrograde expansion. Consecutive only balloon angioplasties were performed to break the vessel walls and empty the intramural haematoma. POBA, plain only balloon angioplasty; NSTEMI, non ST-segment elevation myocardial infarction; ECG, electrocardiogram; SCAD, spontaneous coronary artery dissection; LAD, left anterior descending artery.

Coronary imaging (IVUS or OCT) may be exploited to verify the presence of the guidewire in the true lumen or alternatively using distal microcatheter injection only if the probability of passage into the false lumen is considered low. Imaging is relevant to improve the quality of PCI by reducing the complications but also to optimize treatment by defining the physiopathological mechanism of SCAD (i.e., “inside-out” vs “outside-in”): recent literature data in fact show that angiotypes with confined intramural hematoma given by vasa vasorum hemorrhage are more likely to worsen leading to adverse events [14], therefore an interventional strategy may be preferred. Intracoronary imaging, in particular OCT, may be hampered by difficulty in engaging true lumen, it may also cause SCAD progression and require additional contrast which increases renal damage. When multiple stent placements are necessary, a strategy involving the position of a first stent upstream to the dissection to prevent “squeezing” with retrograde extension of the hematoma may be considered, especially in case of outside-in SCAD (Fig. 4).

A case of successful percutaneous treatment has recently been described using a ‘pull-back injection technique’ for occlusive dissections. It implies wiring the true lumen with a non-hydrophilic wire (to avoid entrance into the false lumen) and to use a stainless steel microcatheter (1.8 Fr) to reach the distal vessel, then an initial tip injection must be done to confirm microcatheter position, finally a vigorous injection (2 mL) must be performed while retrieving the microcatheter to enable connection between true and false lumen and to restore the flux [60].

Likewise, another strategy reported to be applied in cases of occluding SCAD is the antegrade dissection re-entry (ADR) with a StingRay balloon [61]. This technique was initially developed for the treatment of chronic total occlusions (CTOs) and is more complex than the previous one reported (especially in the acute setting): it is based on sub-intimal wiring and positioning of a StingRay balloon which allows withdrawal of the hematoma through the balloon catheter (subintimal transcatheter withdrawal technique, “STRAW technique”).

In addition to the short-term complications of SCAD, a relevant risk of recurrence either in the index coronary segment or in other segments; is due to the tendency of the vessel wall to form new dissections or mural hematomas, due to associated vascular disease (e.g., FMD, chronic inflammatory disease) or predisposing factors not yet identified. Since most SCADs heal within 30 days [27], SCAD recurrence may be defined when it occurs in the same segment at least 30 days after the index event or in another coronary segment even before [13]. The rate of recurrences reported in the literature are dependent on the definition used, follow-up time and therapy. Tweet *et al*. [62] report a recurrence rate of 17% at 47 months and 29.4% at 10 years, the Vancouver group of 10.4% with a mean follow-up of 3 years [41], the Italian-Spanish group reports 6.0% at 1 year [13], Lettieri *et al*. [30] of 4.7% with a mean follow-up of 31 months.

At the current state of knowledge, there is no interventional therapy that can prevent recurrence of SCAD other than rehabilitation/drug therapy.

## 5. Complications

### 5.1 PCI Complications

In SCAD, percutaneous revascularization is hampered by worse outcomes compared to atherosclerotic disease [28, 47, 63], due to the following issues:

- risk of guide placement and subsequent dilatation/stenting within the false lumen.

- risk of “squeezing” of the hematoma with anterograde or retrograde propagation of the dissection.

- risk of under-expansion of the stent by subsequent resorption of the intramural hematoma.

- risk of coronary artery perforation.

Since SCAD is a disease related to the weakening of the arterial wall, patients with this condition often present an intrinsic frailty which leads to an increased risk of iatrogenic dissection. This may occur while entering the true lumen with a guidewire but even in cases of high-pressure injections.

**Fig. 5.**
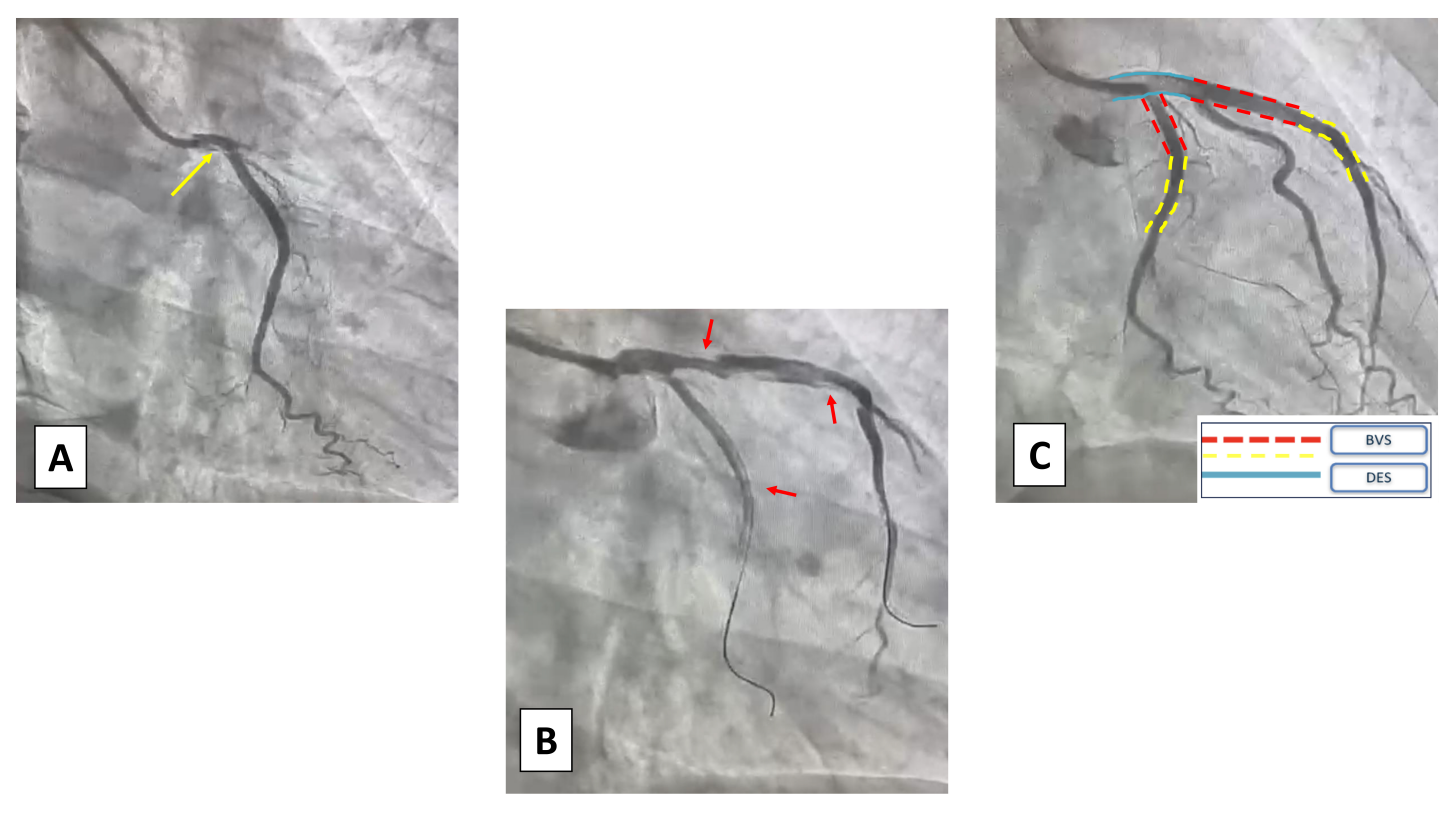
**Case 4: Woman, 64 yo, presenting with an anterior STEMI**. (A) At coronary angiogram, there was evidence of type 1 SCAD of the left main (LM) coronary artery (yellow arrow). (B) Due to the clinical presentation an interventional strategy was chosen and two non-polymeric wires were put in the Cx and LAD, complicated by multiple dissections of the proximal vessels (red arrows). (C) A hybrid strategy of DES+BVS was chosen to avoid full metal stenting. STEMI, acute myocardial infarction with ST-segment elevation; SCAD, spontaneous coronary artery dissection; Cx, circumflex artery; LAD, left anterior descending artery; DES, drug-eluting stent; BVS, bioresorbable vascular scaffolds.

Another common complication is the propagation of the existing dissection which may compromise the clinical presentation of the patient: it may occur during PCI of a shorter tract and forces the operator to apply a longer stenting (Fig. 5).

Furthermore, even when true lumen is appropriately wired, the subsequent implantation of stent may determine the “squeezing” of the intramural hematoma (with possible extension of the dissection itself) and increase the risk of in-stent restenosis and stent thrombosis. Also, the presence of a hematoma may prevent adequate stent expansion so as to cause malapposition upon complete intramural hematoma (IMH) resorption.

Finally, the last common complication regarding the difficulty in wiring the true lumen: in case of wiring and ballooning of the false lumen the dissection may worsen, thus intracoronary imaging may help the operator to avoid procedural mistakes.

### 5.2 Arrhythmic Complications and Implantable Cardioverter Defibrillator (ICD) Implantation

Based on recent data, ventricular tachycardia/ventricular fibrillation (VT/VF) complicate acute SCAD presentation from 4% to 14% of the cases especially during peripartum [4, 41] and the long-term outcomes are currently underknown. Recent observational studies such as Cheung *et al*. [64] have shown that these patients are more likely to have poor in-hospital outcomes, including unplanned revascularization, repeat MI, and heart failure and recurrences of malignant arrhythmias, in particular, both the occurrence of VT/VF and left ventricular ejection fraction (LVEF) <50% at SCAD presentation were identified as independent predictors of post-discharge VT/VF during follow-up.

Nevertheless, the indication for cardiac defibrillator implantation after malignant arrhythmias in SCAD is also a controversial topic [65]. Several studies [66, 67, 68] enhanced no benefit in patients who received an ICD post-SCAD. In these cases, a temporary wearable cardioverter defibrillator (as life-vest) could be appropriate in order to allow recovery of LVEF and to monitor, instead, and a permanent ICD implant, especially in cases of persistent LV dysfunction, should be discussed by a multidisciplinary team [64]. These latter patients could be closely monitored perhaps with an evaluation of scar burden with cardiac magnetic resonance (CMR) that can help stratify the global arrhythmic risk. Mainly since an extensive myocardial scar with a residual impaired LVEF or in cases of potentially higher rates of SCAD recurrence. A subcutaneous device (s-ICD) should be preferred, due to the less invasiveness of the surgery and the lower rate of complications.

As aforementioned, SCAD represents an increasingly frequent cause of ACS due to the greater attention in defining its diagnosis and increased knowledge on the disease. However, the high rates of periprocedural complications, the vascular frailty and the different physiopathology of SCAD patients often leads to multiple concerns on the best way of treatment.

**Fig. 6.**
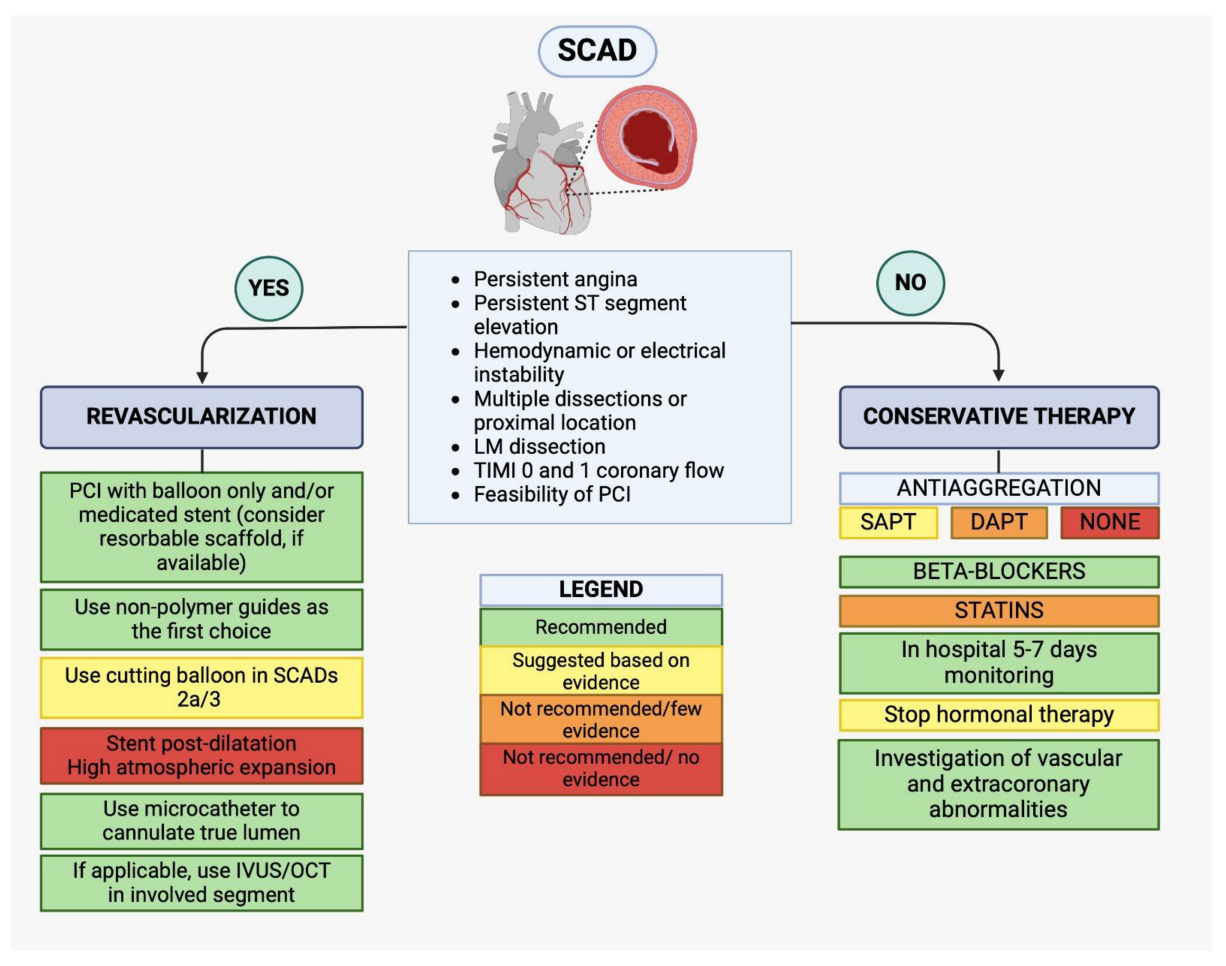
**(Central Figure)—Therapeutic algorithm for SCAD management**. SCAD, spontaneous coronary artery dissection; SAPT, single antiplatelet therapy; DAPT, dual antiplatelet therapy; IVUS, intravascular ultrasound; OCT, optical coherence tomography; LM, left main; TIMI, thrombolysis in myocardial infarction; PCI, percutaneous coronary intervention.

In response to these needs, we propose a therapeutic algorithm, formulated by collecting all available evidence in the literature, on either medical and interventional treatment in the cath-lab. In cases of complicated forms of SCAD, percutaneous revascularization is indicated, if possible, guided by intracoronary imaging (OCT/IVUS). In these cases, PCI with balloon only or stent with reasonable scaffold are recommended instead using high atmosphere post-dilatation is contraindicated. From a technical standpoint the use of a non-polymeric guide is indicated, and also a cutting balloon in case of SCADs 2a/3. But in most cases, a conservative approach is adequate in which statin therapy has not to be included instead the most approved management is the usage of betablockers and single or dual antiplatelet therapy. This is summarized in Fig. 6 (Central Figure).

## 6. Conclusions

SCAD is a rare cause of acute coronary syndrome whose pathophysiology, clinical presentation, diagnosis and treatment have been increasingly studied only in the recent years. In particular, new developments have emerged regarding its treatment in interventional and medical settings, with regard to management in the cath-lab and antithrombotic therapy. For these reasons, participation in dedicated registries and the habit of looking for SCAD on angiography can increase the number of recognized cases, while knowledge of the clinical and management peculiarities of this pathology can help the clinician to set the best treatment program, appropriate follow-up and prevention strategies.
